# Could early life DHA supplementation benefit neurodevelopment? A systematic review and meta-analysis

**DOI:** 10.3389/fneur.2024.1295788

**Published:** 2024-04-05

**Authors:** Ruolan Hu, Juan Xu, Yimin Hua, Yifei Li, Jinrong Li

**Affiliations:** Key Laboratory of Birth Defects and Related Diseases of Women and Children of MOE, Department of Pediatrics, West China Second University Hospital, Sichuan University, Chengdu, Sichuan, China

**Keywords:** DHA, supplementation, offspring neurodevelopment, systematic review, meta-analysis

## Abstract

**Background:**

Docosahexaenoic acid (DHA) plays a crucial role in the growth and functional development of the infant brain. However, the impact of additional DHA supplementation on neurodevelopment in infants remains controversial in randomized controlled trials. In this systematic review and meta-analysis, we aimed to investigate the effects of prenatal and postnatal DHA supplementation on neurodevelopment.

**Methods:**

We systematically searched the MEDLINE, EMBASE, and Cochrane Library electronic databases using a predefined strategy until 8 February 2024. We extracted relevant study characteristics and outcomes related to the nervous system. Two independent reviewers critically evaluated the included studies to assess their validity and risk of bias.

**Results:**

A total of 21 studies met our inclusion criteria, one study was removed after quality assessment, and the meta-analysis included 9 randomized controlled trials. The meta-analysis results indicated that there was no statistically significant difference between the DHA supplementation group and the placebo group, as assessed by the Mental Development Index [MDI; mean difference (MD), 0.41; 95% confidence interval (CI), −0.91 to 1.73; *p* = 0.55]. However, the DHA group had a significantly higher Psychomotor Development Index (PDI) than the placebo group (MD, 1.47; 95% CI, 0.23 to 2.72; *p* = 0.02). Subgroup analyses based on populations showed that DHA supplementation was superior to placebo for infants in both MDI (language score conversion; MD, 2.05; 95% CI, −0.16 to 4.26; *p* = 0.07) and PDI (MD, 1.94; 95% CI, 0.23 to 3.65; *p* = 0.03). Other subgroup analyses indicated no statistical differences between the two groups. The remaining assessments that could not be summarized quantitatively underwent a narrative evaluation.

**Conclusion:**

Based on the BSID assessments, DHA supplementation in infants may have potential neurodevelopmental benefits. Because the meta-analysis included few high-quality articles and had some limitations, more relevant articles are needed to address the need for separate DHA supplementation in infants, pregnant women, and lactating mothers.

**Systematic review registration:**

https://www.crd.york.ac.uk/prospero/display_record.php?ID=CRD42022348100, identifier: CRD42022348100.

## Introduction

Sufficient nutrition supplementation during the prenatal and early postnatal phases plays a crucial role in facilitating programmed and optimal developmental processes. Conversely, malnutrition hinders the normal progression of development and increases the risk of metabolic or cardiovascular diseases later in life. These observations underscore the significance of nutrition supplementation during the peri-pregnancy period (1). It is recommended that infants consume 100 mg of DHA per day during the first year of life (2, 3), while for pregnant women, many authorities and expert scientific organizations recommend an additional 200 mg of DHA per day (4, 5). Several organizations, including the March of Dimes, the American Academy of Pediatrics, and the Food and Agriculture Organization of the United Nations, agree that pregnant and lactating women should have a minimal intake of 200–300 mg of DHA per day (6). The best way to supplement DHA is usually through dietary intake of foods containing DHA, such as deep-sea fish (such as salmon, cod, tuna, etc.), cod liver oil, seaweed, etc. In addition, nutritional supplements containing DHA are also a good choice, such as fish oil or algae oil. While the provision of essential nutrients is widely recognized, the potential benefits of additional nutrition supplementation for pregnant women and postnatal infants remain a subject of ongoing debate (7), particularly concerning elements implicated in neurological development and even academic performance. Inadequate or restricted nutrition intake essential for neuronal growth is associated with cognitive and motor development delays and neurodevelopmental impairments. Nonetheless, further investigation is required to elucidate whether supplementary nutrition interventions can potentially enhance the development of the neurological system.

Among the various essential nutrients, docosahexaenoic acid (DHA, 22:6 omega-3) stands out as a long-chain polyunsaturated fatty acid (LC-PUFA) belonging to the ω-3 (n–3) family. DHA possesses a 22-carbon chain and 6 cis double bonds (22:6*n* – 3) and is highly concentrated in neural tissues. During cerebral development, it has been observed to accumulate in the fetal brain (8–10). Some studies have shown that reduced levels of DHA in the brain have been associated with impaired neurogenesis and neurite growth and alter the metabolism of several neurotransmitters, including dopamine, serotonin, and acetylcholine (11–13), highlighting its crucial role in the functional maturation of the infant’s brain (14). Typically, postnatal DHA is predominantly obtained through maternal breast milk, which serves as the natural and optimal source of infant nutrition and development, offering numerous benefits for maternal health (15). Maternal breast milk naturally contains significant quantities of DHA, ensuring adequate prenatal and postnatal growth. A comprehensive study analyzing breast milk from over 2,400 women reported an average DHA concentration of 0.32/100 g in fatty acids (FAs) (14). Nevertheless, the potential benefits of additional DHA supplementation for developing the neurological system remain uncertain. While specific studies have demonstrated that DHA supplementation enhances cognitive and visual development and function in preterm infants (8), several studies have presented contrasting findings.

Consequently, there is a need to ascertain the potential effects of dietary supplementation with DHA on neurological development in infants. Moreover, it is vital to acknowledge the crucial role of DHA in maintaining neural function (16, 17). However, the existing clinical trials investigating DHA supplementation in infants or pregnant women have been hindered by limitations such as small sample sizes and disparate evaluation systems, preventing the comprehensive synthesis of all relevant findings. Therefore, the present systematic review aims to provide a comprehensive assessment and meta-analysis of the available evidence regarding the efficacy of DHA supplementation during the prenatal and postnatal period, explicitly examining its impact on mental and psychomotor development. The secondary outcomes are the effects of DHA supplementation on visual acuity and cognition in children, which were briefly analyzed.

## Methods

### Protocol

This review complied with the Cochrane Handbook for Systematic Reviews and Interventions recommendations and was recorded according to the PRISMA systematic review guidelines (18). The systematic review protocol was registered in the International Prospective Register of Systematic Reviews (PROSPERO) under registration number CRD42022348100. After researching, the number of cohort studies related to DHA supplementation was small. In contrast, RCT demonstrate significant advantages in establishing causal relationships, controlling confounding variables, and enhancing both internal and external validity of research. Therefore, we ultimately opted to include only RCTs, which differed slightly from from our protocol.

### Search strategy

The systematic search was conducted in three electronic databases: PubMed, EMBASE (Ovid), and Cochrane Library. The search included human-involved studies and was not restricted by publication date. Keywords in the search strategy included [docosahexaenoic acid], [supplements], and [nervous systems] and their synonyms. The entire databases search strategies can be found in Supplementary Appendix 1. The search was updated to the end of February 2024 with language restricted to English. Relevant studies and potential research were searched and reviewed manually.

### Study selection

Citations were collected in a reference manager software program (EndNote X9), and duplicates were eliminated. Citations initially selected by the systematic search were first retrieved as titles and/or abstracts and preliminarily screened. Then, relevant reports were retrieved as complete manuscripts and assessed for compliance with eligibility criteria.

We followed the population-intervention-comparison-outcomes-study design (PICOS) model for defining our eligibility criteria:

1. Population: pregnant women or postpartum nursing mothers without high-risk pregnancy (e.g., psychiatric disorders or pregnancy-induced complications including hypertension, preeclampsia) or infants without any genetic or chromosomal abnormalities.
2. Intervention: DHA supplementation (other polyunsaturated fatty acids were much smaller than that of DHA, e.g., DHA: EPA ≥ 5:1).
3. Comparison: Placebo or no fatty acids supplementation.
4. Outcomes: The outcome evaluation should include any parameters in neurological development: The primary outcome was neurodevelopment, which was explicitly examined using the mental (MDI) and psychomotor development (PDI) indices on Bayley Scale of Infant and Toddler Development (BSID). The secondary outcomes included visual acuity, behavioral development, sleep quality, language skills, cognitive development, and attention variables.
5. Study: randomized, controlled trials; we excluded animal studies conference abstracts or protocols and studies where we could not calculate or obtain missing outcome data.

### Data extraction

Two investigators (Ruolan Hu and Yifei Li) independently assessed the eligibility of reports at the title and/or abstract level, with a supervisor (Jinrong Li) determining the divergences together. Studies that met the inclusion criteria were selected for further analysis. We manually searched the reference lists of included studies, existing systematic reviews and all articles citing the included studies on Google Scholar. Then, relevant reports were retrieved as complete manuscripts and assessed for inclusion and exclusion criteria compliance.

The following data were extracted by two investigators (Ruolan Hu and Yifei Li) using a standardized study form: authors’ names, publication year, study design, follow-up period, location, sample size, gestational age, birth weight, intervention protocol, actual DHA intake, supplementary time, placebo, measurement type, and results. The third investigator (Jinrong Li) cross-checked the information of the included studies.

### Risk of bias assessment

The risk of bias (ROB) of randomized controlled trials was assessed by two independent reviewers using the ROB tool in the Cochrane Handbook 5.1.0 (19). The indicators of ROB include random sequence generation, allocation concealment, blinding of patients and caregivers, blinding of outcome assessment, data completeness, selective outcome reporting, and other biases. Each indicator will be judged as high risk, low risk, or unclear risk for the evaluation result. Two investigators independently conducted all risk of bias assessments, and we resolved any differences in their assessments through team discussion.

### Publication bias

Publication bias was assessed using a funnel plot, a commonly employed method to evaluate potential biases. RevMan 5.4 software was utilized to estimate publication bias for each pooled result in this meta-analysis. The software generated a visual representation, presenting a distribution of individual study results as independent symbols. These representative plots were instrumental in illustrating the potential presence of publication bias. In cases where the figure displayed a symmetric pattern, it indicated the absence of publication bias in the pooled data. Conversely, an asymmetric figure suggested the existence of publication bias.

### Sensitivity analysis

One set of study data was systematically removed to determine whether any single study was incurring undue weight in the analysis. The pooled results for the remaining studies were rechecked to determine whether the results had a significant change. Sensitivity analysis was conducted for every study.

### Data synthesis and analysis

We performed the meta-analysis using RevMan 5.4. We used the mean difference (MD) with 95% CI for continuous data to present the pooled results. Furthermore, the inverse-variance (IV) method was used for continuous outcomes. All statistical methods used were confirmed by a statistician trained in meta-analyses and systematic reviews. We used I2 statistics as a guide to assess heterogeneity, along with a visual inspection of forest plots. I2 > 50% indicated significant heterogeneity across studies (20). To explore the source of heterogeneity, we performed subgroup analyses by the studied population (DHA supplementation during pregnancy, lactation and infant direct supplementation). *p* < 0.05 was considered statistically significant in this trial, except where otherwise specified. If the standard deviations (SDs) were not reported directly, the variances could be imputed from the standard error (SE). For the assessments that could not be pooled, a narrative evaluation was performed to present the results of a systematic review.

## Results

### Study selection

**Figure 1:**
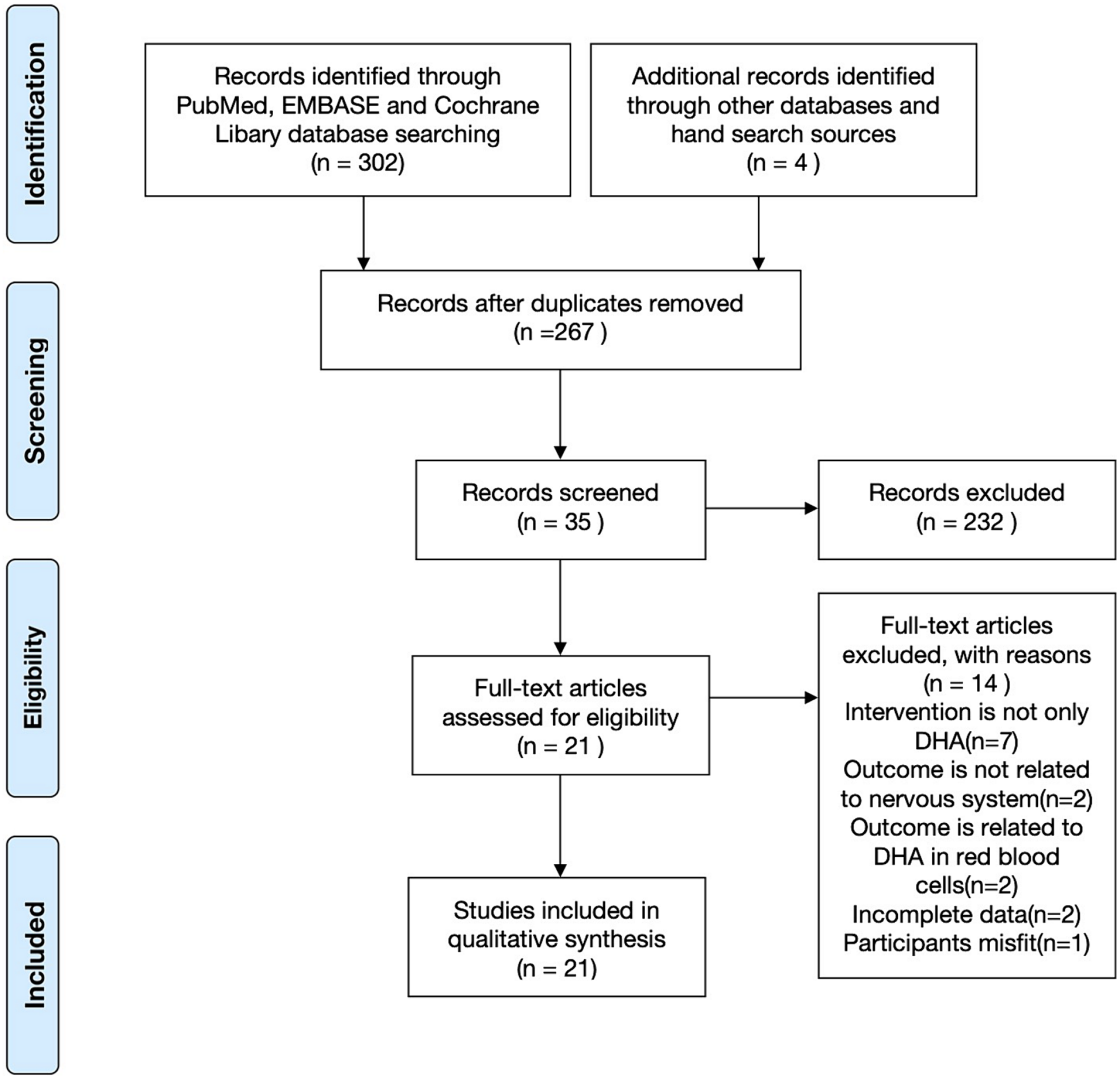
Flow diagram of literature search according to the PRISMA statement.

A total of 304 articles were initially identified through a systematic search, with an additional article obtained through a manual search. After removing duplicates, 267 articles remained and underwent further review. Screening the titles and abstracts resulted in selecting 35 articles considered potential candidates for full-text evaluation. Articles were excluded based on several criteria: (1) not being RCTs, (2) involving subjects other than pregnant women, children, or breastfeeding mothers, (3) utilizing complex supplements with high polyunsaturated fatty acids (except for DHA) content, and (4) lacking neurodevelopmental assessments as part of their outcome evaluations. Ultimately, 21 studies met this systematic review’s rigorous inclusion and exclusion criteria (21–41) (Figure 1).

### Risk of bias

**Figure 2:**
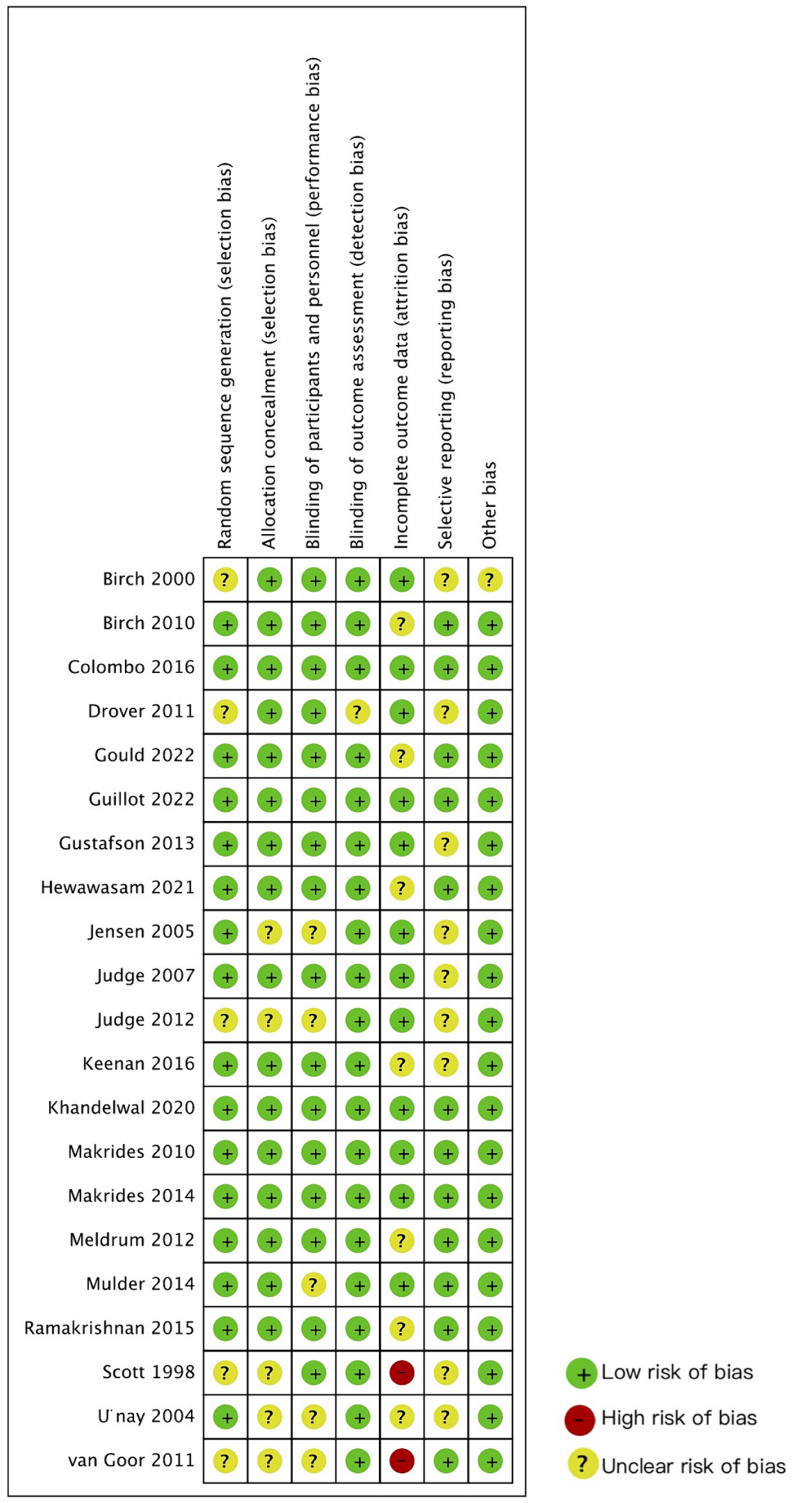
Risk of bias summary: judgments about each risk of bias item for each included study.

Figure 2 summarizes the quality assessment results for the included studies. The majority of the trials were categorized as having an unclear risk of bias. The most common deficiencies identified among the enrolled studies are as follows: nine studies did not provide a research protocol, five studies lacked sufficient information regarding allocation concealment, five studies did not provide blinding information, nine studies had a significant number of participants dropping out, one of them did not mention the reasons of dropping out, five studies did not provide detailed information about the generation of the allocation sequence, and one study reported original data as standard error while the other papers reported standard deviation, which hindered the calculation of meta-analysis.

Overall, according to the criteria of overall quality, 5 of the 21 studies were identified as high quality (26, 27, 36–38, 41) with no items indicating unclear or high risk of bias; 14 studies were evaluated as moderate quality (23, 29–32, 39) with no items at high risk of bias. However, two studies were still considered low quality (22, 24, 25, 28, 42). Although nearly half of the participants dropped out in Van Goor’s study (22), the number of participants who dropped out for each reason in the experimental and control groups was similar. Thus, Van Goor’s study (22) was eligible for the systematic review, while Scott’s study (24) was excluded because its low quality.

### General study characteristics

Finally, the 20 RCTs were included in this systematic review. However, only 5 RCTs utilized the same outcome measurement tool, namely the BSID-II, and 4 RCTs used the BSID-III, which can convert scores to MDI and PDI, thus could be combined for meta-analysis (43–46). Nonetheless, the remaining 12 papers will be included and analyzed in this systematic review. Table 1 provides a summary of the key characteristics of the included studies. All of the studies incorporated in this review were RCTs. The majority of the studies (55%) were conducted in the United States (21, 28–32, 34, 38–41), while others were carried out in Australia (26, 35, 37), Mexico (25), Canada (21, 36), the Netherlands (24), Turkey (23), India (27), New Zealand and Singapore (35, 37). The publication dates of the included studies ranged from 2000 to 2022, indicating the continued interest in DHA administration. Among the 20 studies, 7 employed a postnatal DHA supplementation strategy for infants, 9 described a protocol involving DHA supplementation during pregnancy, 2 provided maternal DHA supplementation after birth, and 2 were maternal DHA supplementation from pregnancy to postpartum. The sample sizes of the included studies varied, ranging from 27 to 863 participants (25, 31). The primary outcomes in the neurodevelopment evaluation were the MDI and PDI based on the BSID. Additionally, secondary outcomes encompassed parameters such as visual acuity, behavioral development, sleep quality, language skills, cognitive development, and attention variables.

**Table 1:** Characteristics of included studies in the systematic-review.

Author	Location	Design	Population	Intervention	Comparator	Results-outcome of interest
**Infant**						
B. Unay 2004	Turkey	RCT	1. full term newborn	Intervention group be fed exclusively with the same formula supplemented with DHA (formula A: 0.5 g/100 g DHA) for the 16-week feeding period.	Comparison group received only a DHA unsupplemented but otherwise similar formula(formula B: 0 g/100 g DHA)	BAEP:
			2. appropriate size for gestational age without breast fed			At the study entry
			(Intervention-28, Control-26)			no significant differences
						At 16 weeks
						The decreases were significantly greater in the formula A than the formula B group (*p* < 0.05)
Drover, J. R. 2011	USA	RCT	1. healthy term (37 to 42 weeks gestation)	Diet group received cow’s milk-based term infant formulas:	Control group received formula with no DHA or ARA from 1–9 days to 12 months after birth	BSID II:
				0.32% DHA with 0.32% fatty acids from DHA (17 mg/100 kcal);		
			2. formula-fed singleton births with birthweight appropriate-for-gestational-age (2,490 to 4,200 g) were included in the trial.	0.64% DHA (34 mg/100 kcal);		There were no diet group differences on the Mental Development Index (MDI), the Psychomotor Development Index (PDI), or the Behavior Rating Scale (BRS) of the BSID II.
			117 children (28–32 per diet group)	0.96% DHA (54 mg/100 kcal).		However, when three DHA-supplemented formulas were combined and compared to control children, a significant difference emerged: the MDI scores of DHA-supplemented children were higher (104.1 v. 98.4; *p* = 0.02).
Birch, E. E. 2010	USA	RCT	1. Healthy, term, formula-fed, singleton-birth infants	DHA group received DHA with 0.32% fatty acids from DHA (17 mg/100 kcal), and the experimental formulas with 0.64% DHA (34 mg/100 kcal) or 0.96% DHA (51 mg/100 kcal). All DHA-supplemented formulas provided 0.64% fatty acids as ARA (34 mg/100 kcal).	Control group received formula with 0% DHA until the first 12 months of life.	Visual acuity:
						At 12 months of age
			2. 37–42 weeks’ gestation; 2,490–4,200 g birth weight			Infants fed control formula had significantly poorer visual evoked potential visual acuity than did infants who received any of the DHA-supplemented formulas (*p* < 0.001). There were no significant differences in visual evoked potential visual acuity between the 3 amounts of DHA supplementation for either site at any age tested.
Birch, E. E. 2000	USA	RCT	1. health term infants (born at 37 to 40 weeks postmenstrual age)	DHA group received formula supplemented with 0.35% DHA (of total fatty acids).	Control group received formula without DHA.	BSID-II:
						At 18 months of age
			(Intervention-17, Control-20)	Nearly 131 mg/d		Both the cognitive and motor subscales of the MDI showed a significant developmental age advantage for DHA− and DHA+ AA-supplemented groups over the control group. While a similar trend was found for the language subscale, it did not reach statistical significance. Neither the Psychomotor Development Index nor the Behavior Rating Scale of the BSID-II showed significant differences among diet groups.
Meldrum, S. J. 2012	Australia	RCT	1. term infants	DHA group received a high-dose DHA-enriched ethyl ester FO supplement, aimed at delivering 250–280 mg DHA/d.	Control group received an image-matched placebo containing olive oil.	Child Behavior Checklist:
						at 18 months
						There was a significant difference between the groups for anxious/depressed behaviors, with more children in the FO group scoring while no significant differences between the groups in any of the remaining CBCL subscales.
						MCDI:
						at 12 and 18 months
						Children in the FO group performed significantly better in language assessments at 12 and 18 months of age with higher percentile ranks of both later developing gestures (P 1/4 0·007; P 1/4 0·002, respectively) and the total number of gestures (P 1/4 0·023; P 1/4 0·006, respectively) compared with placebo.
						BSID-III:
						at 18 months
			(Intervention-218, Control-202)			There was no significant difference between the groups in the standard or composite scores.
Hewawasam, E. 2021	Australia, New Zealand and Singapore	RCT	1. preterm infants from the N3RO trial (born before 29 weeks’ gestation)	DHA group received an enteral emulsion that provided 60 mg of DHA per kilogram of body weight per day until 36 weeks’ post menstrual age. (It is estimated to be DHA 60 mg/d, and later increases with weight gain.)	Control group received no DHA (soya oil—control) emulsion from the first 3 days of enteral feeds until 36 weeks of postmenstrual age or discharge home, whichever occurred first.	Attention:
						at 18 months
						There was no evidence of a difference between groups in the latency of distractibility (adjusted mean difference: 0.08 s, 95% CI −0.81, 0.97; *p* = 0.86).
						Bayley-III:
						at 18 months
			(Intervention-241, Control-239)			The cognitive score in the Bayley-III assessment did not significantly differ between the DHA and control groups (adjusted mean difference −0.93, 95% CI −7.13, 5.28; *p* = 0.77). Similarly, the motor and language scores did not significantly differ between the treatment groups in either unadjusted or adjusted analyses.
Gould, J. F. 2022	Australia, New Zealand and Singapore	RCT	1. preterm infants from the N3RO trial (born before 29 weeks’ gestation)	DHA group received an enteral emulsion that provided 60 mg of DHA per kilogram of body weight per day until 36 weeks’ post menstrual age. (It is estimated to be DHA 60 mg/d, and later increases with weight gain.)	Control group received no DHA (soya oil—control) emulsion from the first 3 days of enteral feeds until 36 weeks of postmenstrual age or discharge home, whichever occurred first.	WPPSI:
						At 5 years of age
			(Intervention-241, Control-239)			The mean (±SD) FSIQ scores were 95.4 ± 17.3 in the DHA group and 91.9 ± 19.1 in the control group (adjusted difference, 3.45; 95% confidence interval, 0.38 to 6.53; *p* = 0.03)
						The percentages of children with a clinical diagnosis of autism spectrum disorder, attention deficit-hyperactivity disorder, or other behavioral or neurologic disorders were similar in the two groups.
**Pregnancy**						
Judge, M. P. 2007	USA	RCT	1. pregnant women	DHA group received the DHA-containing cereal-based bars [(300 mg DHA as low EPA fish oil: EPA:DHA, 1:8, per 92 kcal bar) from 24 weeks of pregnancy to delivery]. 214 mg/d average	Placebo group received the cereal-based placebo bars containing corn oil	Visual acuity scores (mean ± SD):
						Four-month
						I: 3.7 ± 1.3 cycles/degree
						C: 3.2 ± 1.3 cycles/degree
						(*p* = 0.018)
						Six-month
			2. 18–35 years of age and < 20 weeks’ gestation			I: 5.9 ± 1.2 cycles/degree
			(Intervention-16, Control-14)			C: 5.4 ± 1.3 cycles/degree
Makrides, M. 2014	Australia	RCT	1. Pregnant women	DHA group were asked to consume three 500 mg/d capsules of DHA-rich fish oil concentrate, providing 800 mg/d of DHA and 100 mg/d of eicosapentaenoic acid until delivery.	Control group were asked to take three 500-mg/d vegetable oil capsules without DHA.	GCA scores:
						At 4 years of age
						Mean GCA scores neither differed between groups (adjusted mean difference, 0.29 [95% CI, −1.35 to 1.93], *p* = 0.73), nor did the percentage of children with delayed or advanced GCA scores.
			2. with singleton pregnancies at less than 21 weeks’ gestation.			Other objective assessments of cognition, language, and executive functioning also did not differ between groups.
			(Intervention-313, Control-333)			However, the DHA group had poorer scores on some parentally reported scales of executive functioning and behavior.
Judge, M. P. 2012	USA	RCT	1. healthy pregnant women (18–35 years of age)	Intervention group received DHA containing cereal bars (300 mg DHA) at 24 weeks of gestation until delivery. 214 mg/d average	Comparison group received placebo bars contained corn oil	On postnatal day 1
						Infants of mothers in intervention group had significantly fewer Arousals in Quiet Sleep [*t*_(44)_ = 2.17, *p* < 0.05] and Arousals in Active Sleep [*t*_(44)_ = 2.21, *p* < 0.05] compared to infants born to comparison group.
			2. primiparous or had not been pregnant for the past 2 years			On postnatal day 2
			(Intervention-27, Control-21)			Infants of mothers in intervention group had significantly fewer Arousals in Quiet Sleep [*F*_(46, 1)_ = 5.72, *p* < 0.05] than the comparison group
Gustafson, K. M. 2013	USA	RCT	1. pregnant women	Intervention group received 3 capsules a day, each capsule contained 500 mg of oil from algal oil as a source of DHA (200 mg of DHA per capsule for a total of 600 mg DHA/day) until delivery.	Comparison group received three placebo capsules containing 50% soy and 50% corn oil	NBAS:
			2. between 16 and 35.9 years of age and carrying a singleton pregnancy between the 12th and 20th week of gestation			Intervention group showed significantly higher (i.e., more optimal) scores on the Motor [*t*_(25)_ = 1.87, *p* = 0.038] and Autonomic clusters [*t*_(25)_ = 1.99, *p* = 0.029], and differences approached significance on the Orienting cluster [*t*_(25)_ = 0.55, *p* = 0.092].
			(Intervention-15, Control-12)			
Mulder, K. A. 2014	Canada	RCT	1. pregnant women	DHA group received algal oil triglycerides until delivery.	Placebo group received an equivalent amount of corn and soybean oil.	Visual acuity:
						Infants in the placebo group were at increased risk of not achieving a visual acuity ≥ 3.3 cycles/degree at 2 month of age (OR, 2.50, CI 1.02–6.14, *n* = 184, *p* = 0.03), with no evidence of increased risk of failure to achieve high visual acuity at 12 month of age (OR, 1.23, CI 0.61–2.49, *n* = 176, *p* = 0.35)
						Problem-solving:
						At 9 months of age
						there was no difference in success between the placebo and DHA groups when stratified by sex for infant girls (*p* = 0.21) or boys (*p* = 0.27).
						Similarly, we found no difference in ability to discriminate the non-native language consonant between placebo and DHA groups.
			2. 16 weeks’ gestation and expected to deliver one infant at full-term gestation, with no maternal or fetal complications.	400 mg/d DHA		BSID-III and CDI
			(Intervention-132, Control-138)			Infants in the placebo group were at increased risk of not performing in the highest 25% of infants for words understood (OR 3.22, CI 1.49–6.94, *p* = 0.002) and produced (OR 2.61, CI 1.22–5.58, *p* = 0.01) at 14 month of age, and for words understood (OR 2.77, CI 1.23–6.28, *p* = 0.03) and sentences produced (OR 2.60, CI 1.15–5.89, *P* = 0.02), with a similar trend for words produced (*p* = 0.07) at 18 month of age. Infants in the placebo group were also at increased risk of not performing in the highest 25% of infants on the BSID-III receptive language (OR 2.23, CI 1.08–4.60, *P* = 0.03) and expressive language scales (OR 1.89, CI 0.94–3.83, *p* = 0.05) at 18 months of age.
Makrides, M. 2010	Australia	RCT	1. pregnant women	DHA group were asked to consume three 500 mg/d capsules of DHA-rich fish oil concentrate, providing 800 mg/d of DHA and 100 mg/d of eicosapentaenoic acid until delivery.	Control group were asked to take three 500-mg/d vegetable oil capsules without DHA.	BSID-III
			2. women with singleton pregnancies at less than 21 weeks’ gestation.			At 18 months of age
			(Intervention-351, Control-379)			Mean cognitive scores of children from DHA group did not differ from mean scores of children from the control group, although fewer children from the DHA group had cognitive scores indicating delayed cognitive development compared with controls. Overall, mean language scores also did not differ between groups; Motor development, social–emotional behavior, and adaptive behavior did not differ between groups.
Ramakrishnan, U. 2015	Mexico	RCT	1. pregnant women	DHA group received 2 capsules of 200 mg of DHA or placebo from weeks 18 to 22 of gestation through delivery. 400 mg DHA/d	Control group received placebo pills contained a mix of corn and soy oils.	BSID-II:
			2. gestation week 18–22, age 18–35 years and planned predominant breastfeeding for at least 3 months.			At 18 months of age
			(Intervention-365, Control-365)			Intent to treat analysis showed no significant differences by treatment group for the MDI, PDI or BRS.
Colombo, J. 2016	USA	RCT	1. pregnant women in the Kansas University DHA Outcomes Study pregnancy trial	DHA group received either 3 capsules/d of an orange-flavored marine algae-oil source of DHA (200 mg DHA/capsule). 600 mg DHA/d	Control group received 3 capsules containing half soybean and half corn oil (placebo, also orange-flavored).	Tests of visual habituation:
			2. exclude infants born <34 weeks’ gestation			at 4, 6, and 9 months of age
			(Intervention-120, Control-100)			Prenatal maternal DHA supplementation conferred advantages for the infants on attentional tasks (SA and behavioral state) during the first year of life.
Keenan, K 2016	USA	RCT	1. African American pregnant women	DHA group received Control group received two strawberry flavored gel capsules providing: 450 mg of DHA; 40 mg of DPA and ETA; 90 mg EPA; and 10 mg Vitamin E until delivery. 450 mg DHA/day	Control group received a soybean oil placebo contained 990 mg of soybean oil, 16.5 mg Vitamin E, and 10 mg of EPA and DHA for flavor matching purposes.	BSID-III:
			2. at 16–21 weeks of gestation			At 3 months
			(Intervention-43, Control-21)			None of the scores on the BSID-III differed as a function of active supplement vs. placebo.
**Pregnancy + Mother**						
Khandelwal, S. 2020	India	RCT	1. Healthy pregnant women	DHA group received 400 mg/d algal DHA until 6 months postpartum.	Control group received placebo from ≤20 weeks of singleton gestation through 6 months postpartum.	DAS II:
			2. 18–35 years; ≤20 weeks single gestation; with no medical complications or chronic diseases			At 12 months
			(Intervention-433, Control-430)			The mean development quotient (DQ) scores in the DHA and placebo groups were not statistically significant (96.6 ± 12.2 vs. 97.1 ± 13.0, *p* = 0.60).
van Goor, S. A. 2011	Netherlands	RCT	1. healthy women	Intervention group received 220 mg DHA and 1 capsule containing soy bean oil every day from enrollment until 3 months after delivery.	Comparison group received 2 capsules containing soy bean oil	The NOS, the fluency score, the prevalence of simple and complex MND as well as the Bayley MDI and PDI scores did not differ between the groups.
			2. between the fourteenth and twentieth weeks of pregnancy			
			(Intervention-41, Control-34)			
**Mother**						
Jensen, C. L. 2005	USA	RCT	1. mothers	DHA group received 1 capsule (contained a high-DHA algal triacylglycerol) daily for 4 months, starting within 5 day after delivery. 200 mg DHA/d	Control group received control capsule contained a 50:50 mixture of soy and corn oils.	Gesell Developmental Inventory (motor):
						At 12 months of age
						At 30 months of age
						Neither the neurodevelopmental indexes of the infants at 12 months of age nor the visual function at 4 or 8 months of age differed significantly between groups.
			2. age between 18 and 40 years and her infants gestational age ≥ 37 weeks, and infant birth weight between 2,500 and 4,200 g.			BSID-II:
			(Intervention-88, Control-83; 12 months)			At 30 months of age
			(Intervention-83, Control-77; 30 months)			Psychomotor Development Index of the supplemented group was higher (*P* < 0.01) at 30 months of age.
Guillot, M. 2022	Canada	RCT	1. lactating mothers	DHA group received high dose of DHA (i.e., 1.2 g DHA daily to achieve ~1% of DHA in breast milk).	Control group received placebo capsules within 72 h of delivery until 36 weeks’ postmenstrual age.	BSID-III:
						at 18 to 22 months’ corrected age
			2. delivered before 29 weeks’ gestation			The mean differences in Bayley-III between children in the DHA and placebo groups were −0.07 (95% CI −3.23 to 3.10, *p* = 0.97) for cognitive score, 2.36 (95% CI −1.14 to 5.87, *p* = 0.19) for language score, and 1.10 (95% CI −2.01 to 4.20, *p* = 0.49) for motor score.
			(Intervention-234, Control-223)			Neonates born <27 weeks’ gestation exposed to DHA performed better on the Bayley-III language score, compared with the placebo group (MD 5.06, 95% CI 0.08–10.03, *p* = 0.05).

### MDI and PDI

**Figure 3:**
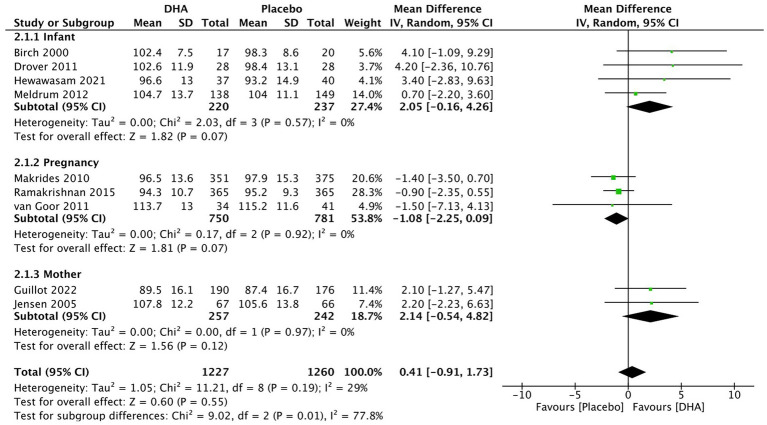
Forest plot summarizing the effect of DHA supplement on infants’ neurodevelopment through MDI (language scores conversion).

Nine studies were included in the assessment of MDI and PDI scores, based on the BSID, to evaluate the impact of DHA supplementation on neurodevelopment. Four of these studies applied BSID-III language score conversion to include meta-analyses of mental development (43). The overall pooled results for MDI assessment (Figure 3) indicated no statistically significant difference (MD = 0.41, 95% CI −0.91 to 1.73, I2 = 29%, *p* = 0.55). Subgroup analysis based on the administration of DHA postnatally to infants (MD = 2.05, 95% CI −0.16 to 4.26, I2 = 0%, *p* = 0.07), during maternal pregnancy (MD = −1.08, 95% CI −2.25 to 0.09, I2 = 0%, *p* = 0.07), and to breastfeeding mother (MD = 2.14, 95% CI −0.54 to 4.83, I2 = 0%, *p* = 0.12) also failed to reveal a significant difference. The results of converting cognitive scores to MDI by applying Lowe et al.’s (45) conversion formula [Bayley-III score = (0.59 X Bayley II) + 52] were presented in Supplementary Appendix 2. Nevertheless, the DHA supplementation to infants differed significantly from the control group (MD = 3.80, 95% CI 0.58 to 7.03, I2 = 0%, *p* = 0.02). Other subgroups [during pregnancy (MD = −0.72, 95% CI –1.99 to 0.55, I2 = 0%, *p* = 0.26)] and mother (MD = 0.89, 95% CI −2.48 to 4.26, I2 = 0%, *p* = 0.60) showed no significant difference between the experimental group and control group. Likewise, there was no statistical difference overall (MD = 0.16, 95% CI −1.08 to 1.40, I2 = 5%, *p* = 0.80).

**Figure 4:**
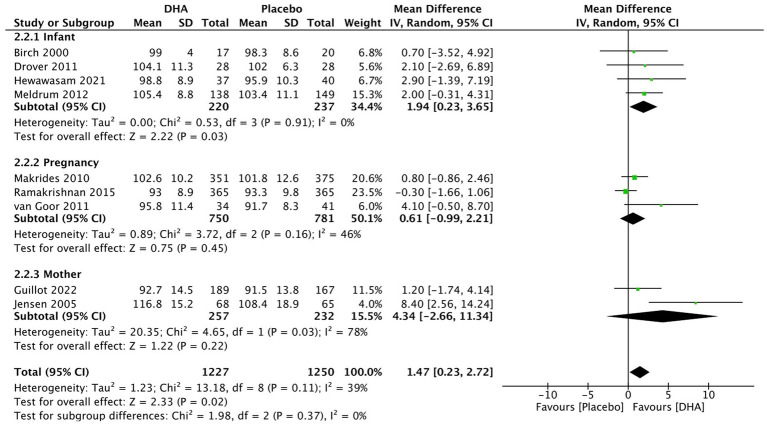
Forest plot summarizing the effect of DHA supplement on infants’ neurodevelopment through PDI.

For PDI assessment (Figure 4), the overall pooled results demonstrated a statistically significant difference (MD = 1.47, 95% CI 0.23 to 2.72, I2 = 39%, *p* = 0.02). DHA administration to postnatal infants (MD = 1.92, 95% CI 0.23 to 3.65, I2 = 0%, *p* = 0.03) positively affected on PDI evaluation. However, other two subgroups analysis indicated that DHA supplementation administered during maternal pregnancy (MD = 0.61, 95% CI −0.99 to 2.21, I2 = 46%, *p* = 0.45) and to breastfeeding mother (MD = 4.34, 95% CI −2.66 to 11.34, I2 = 78%, *p* = 0.22) did not result in a significant difference.

We divided the studies on infant DHA supplementation into two subgroups: term infants and preterm infants, for meta-analysis (Supplementary Appendix 3). The results indicated DHA group had significantly higher scores (MDI through cognitive score conversion) than the placebo group among full-term infants (MD = 4.10, 95% CI 0.68 to 7.52, I2 = 0%, *p* = 0.02). However, there were no significant differences between DHA and placebo groups in other outcomes whether premature or term infant. Separate forest plots corresponding to each subgroup analysis were shown in Supplementary Appendix 3.

### Visual development

Four studies were conducted to investigate the effectiveness of DHA supplementation on visual acuity during early life, utilizing different methodologies such as Sweep VEP and Teller Acuity Cards. Due to the variations in data formats, the reported results could not be combined for meta-analysis. Jensen et al. (30) reported no significant differences in VEP between the DHA intervention and control groups at 4 months. However, Judge et al. (28) found that DHA supplementation during pregnancy improved offspring visual acuity at 4 months (*p* = 0.018), although this improvement was not sustained at 6 months. Similar findings were observed in assessments using Teller Acuity Cards. Mulder et al. reported that infants receiving DHA supplementation had a reduced risk of delayed visual acuity development, achieving 3.3 cycles/degree based on Teller Acuity Cards assessment at 2 months (OR 2.50, 95% CI 1.02–6.14, *p* = 0.03). However, DHA supplementation did not significantly improve visual acuity at 12 months (OR 1.23, 95% CI 0.61–2.49, *p* = 0.35) (21).

Colombo et al. (38) showed that DHA supplementation did not affect look duration or habituation parameters (visual attention) across the first year.

Birch et al. (40) reported that infants fed control formula had significantly poorer visual evoked potential visual acuity at 12 month of age than infants who received the DHA-supplemented formulas (*p* < 0.001). There were no significant differences in visual evoked potential visual acuity between the three amounts of DHA supplementation (0.32% DHA, 0.64% DHA, 0.96% DHA) for either site at any age tested which indicated higher amounts of DHA supplementation were not associated with additional improvement of visual acuity.

The articles mentioned above on visual demonstrate the beneficial effects of DHA supplementation for visual acuity in pregnant women (21, 28), while Colombo’s study (38) showed DHA supplementation did not affect visual attention. Similar results were observed in full-term infants receiving DHA supplementation (40). However, no apparent benefits were observed when supplementing mothers with DHA (30).

### Auditory function

In all groups, Unay et al. (15) reported significant decreases in absolute wave and interpeak latencies (measured using brainstem auditory evoked potential) at 16 weeks of age. However, these reductions were more pronounced in healthy, full-term newborns who received DHA supplementation than those who did not (*p* < 0.05).

### Language skills

Seven studies reported the results of language skill measurement. Among them, four studies used the BSID scale (26, 33, 35, 36), while one study employed Clinical Evaluation of Language Fundamentals Preschool (CELF-P2) (41). These studies demonstrated no statistical difference in language skill evaluation at 12, 18, and 48 months. However, Meldrum et al. (33) conducted a study utilizing the Macarthur–Bates Communicative Development Inventory and found that term infants who received high-dose DHA-enriched diets exhibited significantly better performance in language assessments at 12 and 18 months of age. Compared to the placebo group, these children demonstrated higher percentile ranks in both later-developing gestures (*p* = 0.007; *p* = 0.002, respectively) and the total number of gestures (*p* = 0.023; *p* = 0.006, respectively). Furthermore, another study reported that children who received DHA supplementation showed enhanced language development in terms of words understood (OR 3.22, 95% CI 1.49–6.94, *p* = 0.002; OR 2.77, 95% CI 1.23–6.28, *p* = 0.03) and sentences produced (OR 2.61, 95% CI 1.22–5.58, *p* = 0.01; OR 2.60, 95% CI 1.15–5.89, *p* = 0.02) at 14 and 18 months, respectively, as assessed by the McArthur Communicative Developmental Inventory (21).

There did not appear to be a consistent outcome regarding the benefits of DHA supplementation among infants, pregnant women, and mothers for language development.

### Cognitive development

Four studies (26, 33, 35, 36) used the BSID-III scale to evaluate cognitive development. None of the studies’ cognitive composite scores differed between children in the DHA and placebo groups.

Similar to language development, there is no consensus on the benefits of DHA supplementation in infants, pregnant women, and mothers for cognitive performance.

### Behavior

The study conducted by Gustafson et al. (31) revealed significantly higher scores in the motor [*t*_(25)_ = 1.87, *p* = 0.038] and autonomic clusters [*t*_(25)_ = 1.99, *p* = 0.029] based on the Neonatal Behavioral Assessment Scale (NBAS) among children whose mothers received DHA supplementation during pregnancy compared to those who received a placebo. Meldrum et al. reported that term infants who received high-dose DHA had higher scores, indicating a reduced risk of psychological disorders for anxious/depressed behaviors, as assessed through the Achenbach Child Behavior Checklist (33). What’s more, Gould et al. (37) observed that the percentages of preterm infants with a clinical diagnosis of autism spectrum disorder, attention deficit–hyperactivity disorder, or other behavioral or neurologic disorders were similar in the two groups. Furthermore, Makrides et al. (41) also revealed that autism and hyperactivity disorders did not differ between groups.

Supplementing full-term infants and pregnant women with DHA appeared to reduce the risk of psychological disorders, whereas supplementation for preterm infants does not.

### Executive function

Judge et al. (21) demonstrated that infants whose mothers consumed 214 mg/day of DHA during pregnancy experienced significantly fewer arousals in quiet sleep [*t*(44) = 2.17, *p* < 0.05] and active sleep [*t*(44) = 2.21, *p* < 0.05] compared to infants who did not receive DHA on the first postnatal day. Furthermore, ANCOVA analysis indicated that neonates in the DHA group (supplementation for pregnancies) continued to exhibit significantly fewer arousals in quiet sleep on the second postnatal day. Makrides et al. (41) indicated that the DHA group had poorer scores on some parentally reported scales of executive functioning and behavior at 4 years of age. However, the differences were small and unlikely to be clinically significant because all measures were within the normal range.

### Attention

Colombo et al. (38) reported that prenatal maternal DHA supplementation conferred advantages for the infants on attentional tasks (sustained attention and behavioral state) during the first year of life (at 4, 6, and 9 months of age). Nevertheless, Hewawasam et al. (35) showed no evidence of a difference between groups in preterm infant of the latency of distractibility (adjusted MD: 0·08 s, 95% CI −0·81, 0·97; *p* = 0·86) at 18 months of age.

### Publication bias

**Figure 5:**
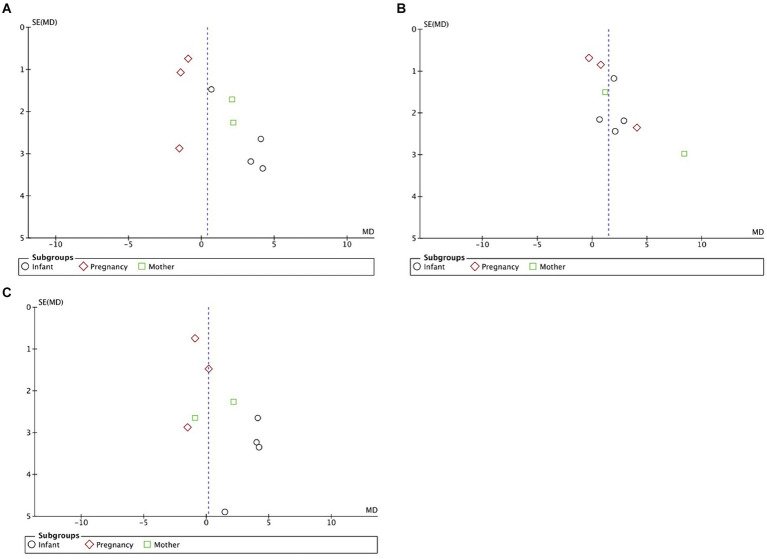
**(A)** Funnel plots detailing publication bias in the selected studies of DHA supplementation’s effect on infants’ MDI (language scores conversion). **(B)** Funnel plots detailing publication bias in the selected studies of DHA supplementation’s effect on infants’ PDI. **(C)** Funnel plots detailing publication bias in the selected studies of DHA supplementation’s effect on infants’ MDI (cognitive scores conversion).

Visual examination of the funnel plots (Figures 5A–C) revealed slight to moderate asymmetry, indicating the possibility of some publication bias across all outcomes. These findings suggest that while no obvious publication bias was observed, it cannot be completely excluded as a potential influence on the present meta-analysis. However, given the limited amount of data included, it is essential to acknowledge that publication bias cannot be definitively ruled out as a factor impacting the results of this meta-analysis.

### Sensitivity analysis

During the sensitivity analysis, the impact of individual studies on the overall effect estimate was assessed by sequentially removing one study at a time and reanalyzing the remaining studies. It was observed that no single study had a substantial influence on the pooled effect estimate when comparing the results of the present systematic review with the meta-analysis. This indicates the robustness and reliability of the findings obtained in this study.

## Discussion

DHA plays a crucial role in developmenting a healthy brain (47). The human brain exhibits significantly higher DHA levels than eicosapentaenoic acid (EPA) (48). Extensive research has shown that DHA supplementation during pregnancy and lactation and the fortification of infant food with DHA leads to increased DHA levels in infant tissues and improved neurological and visual development (49). In addition, the impact of DHA deficiencies on cognitive functions has been extensively studied based on animal models. DHA deficiency can lead to synaptic plasticity, region-specific brain developmental disorders, and an increased risk for neurocognitive impairment (50). Opinions on DHA supplementation have been changing. Until 1998, expert panels of the FDA in the United States and working groups of Canadian authorities did not recommend the inclusion of LC-PUFAs, including DHA, in infant formulas. However, in 2021, the European Commission revised its recommendation on DHA from an optional ingredient to a mandatory nutrient (51). Additionally, there has been a significant expansion in the global omega-3 market, particularly DHA, due to the rising demand for DHA as a vital component of infant formula and supplements (52). Despite the growing usage of DHA, no meta-analysis currently assesses the potential benefits of DHA supplementation alone in the development of the infant nervous system. Therefore, it is imperative to investigate whether infants can benefit from DHA supplementation address this important knowledge gap.

Regarding the primary outcomes, the meta-analysis revealed that DHA supplementation did not benefit mental development. This finding remained consistent across all subgroup analyses, except for DHA supplementation to infants. Moreover, the psychomotor development did significantly differ between the two groups. A discussion according to the subgroup follows.

### Supplementation for infants

The meta-analysis results indicated the potential benefits of DHA supplementation for infants in mental development when given to postnatal infants (cognitive scores conversion). However, we need to note that the MDI of applying language conversion did not show a difference between DHA and placebo groups, indicating that different conversion formulas had heterogeneity. Similar to the total effect, the DHA group of infants had higher psychomotor development scores. The PDI in BSID-II primarily assessed gross and fine movements in infants and young children, whereas the Motor Scale in the Bayley-III assesses gross, fine movements, and hand-eye coordination, and thus may be somewhat heterogeneous. We hoped that more accurate conversion methods between BSID-II and BSID-III would be available in the future. Supplementation of DHA to infants after birth appeared to have mental and psychomotor benefits. For secondary outcomes, we noticed that most studies demonstrated some short-term positive effects of DHA supplementation on infant visual acuity and attention immediately after birth. In contrast, medium-term follow-up results showed no significant differences between the DHA supplementation and control groups. A recent study also demonstrated that higher dietary DHA intake during the first year was significantly associated with better cognitive function at 12 months and improved motor function at 12 months of corrected age, but not at 24 months in very low birth weight infants (53).

### Supplementation for pregnant women

In the subgroup analysis of DHA supplementation for pregnant women, neither MDI nor PDI differed between the DHA and placebo groups. This finding was also shown in cognitive and language scales; Combined with the results of DHA supplementation in infants, the observed benefits of DHA supplementation appear to be time-dependent, with DHA promoting neurological development and maturation in the early stages but not improving neurological function once the neurons have matured. For example, Makrides’ four-year follow-up showed that the differences observed at 18 months (including cognitive delay and mean language scores) were no longer present at 4 years of age (41). This suggests that DHA may facilitate the differentiation and proliferation of neural stem cells but does not contribute significantly to the functional maintenance of mature neural cells. After the maturation stage of the neurological system, specific training becomes more critical for an infant’s neurodevelopment. Moreover, in the long-term follow-up, many other factors would implicate neurodevelopment, alleviating the positive effects of DHA supplementary.

### Supplementation for lactating mother

Maternal DHA supplementation was mentioned in only a few articles. This meta-analysis showed no benefit of maternal DHA supplementation only after delivery on mental and psychomotor development in their offspring. However, the results of the two articles included in the meta-analysis suggested that PDI of the supplemented group was higher at 30 months of age and infants exposed to DHA performed better on the Bayley-III language score, compared with the placebo group.

In addition to the participants of DHA supplementation, there are other factors that affect infant neurodevelopment. Preterm infants are known to be more deficient in DHA than full-term newborns (6), and DHA supplementation in preterm infants seems to be essential. Of the included studies, three (35–37) explicitly mentioned preterm infants, two (36, 37) of which measured different indicators at different points in time for the same group of participants. There were no differences in cognitive, language, motor scale and attention between the DHA and placebo group, while subgroup analysis in Guillot et al. (36) study suggested a potential benefit for language in preterm neonates born before 27 weeks gestational age. The DHA intake in Guillot’s (36) (1.2 g/day) study was much higher than that in the other two studies (35, 37) (60 mg/kg/day), indicating that preterm infants need large amounts of DHA supplementation. What’s more, the dose of DHA supplementation is an important factor. As the recommended supplementation dose of DHA mentioned in introduction, we defined the high dose of DHA directly supplementing infants ≥ 100 mg/day and to pregnant women and lactating women ≥ 300 mg/day. Most studies have applied high doses of DHA supplementation, and there is no consensus on whether DHA improves neurodevelopment. However, Some studies indicated that certain concentrations of DHA can be beneficial for neurodevelopment, and higher concentrations did not confer additional benefit (32, 35, 40). Only two studies (same participants) supplemented low doses of DHA for infants initially, one indicated that using an enteral DHA emulsion until 36 weeks of postmenstrual age was associated with modestly higher FSIQ scores at 5 years of age than control feeding. We conjectured it was because the participants were preterm infants. For pregnant women and lactating mothers, high doses of DHA did not significantly benefit over low doses for the BSID measurement of neurodevelopment in infants, but high dose of DHA appeared to be beneficial for visual acuity. While higher DHA doses (800 mg/day, which was the highest dose of DHA supplementation for pregnant women) might lead to poorer scores on some parentally reported scales of executive functioning and behavior in 4-year-olds (41), the DHA group did not show any difference at 18 months (26).

Based on our results, supplementation for infants with DHA is effective for their neurodevelopment. However, this only shows a significant advantage in PDI, and the results differ with different conversion methods. Therefore, more high-quality evidence is needed to confirm the benefit of DHA supplementation in infants and young children, and we cannot definitively decide whether DHA supplementation should be given in infants. There are few studies on DHA supplementation in preterm infants, and we cannot draw a firm conclusion. For pregnant women, our results were inconsistent, although DHA intake during pregnancy is important to ensure that the fetus obtains an adequate level. A study in this meta-analysis yielded negative results regarding the effectiveness of high-dose DHA supplementation for pregnancies, so further research is warranted to explore this topic in greater depth. The need of DHA supplementation for lactating mother is uncertain.

This review has several notable limitations that should be acknowledged. Firstly, the included studies exhibited significant heterogeneity in participant demographics (encompassing postnatal infants, pregnant mothers, and breastfeeding mothers) and differences in assessment tool editions (BSID-II and BSID-III). Secondly, there was considerable variability in the DHA dosages administered across the studies included in our meta-analysis, and the precise intake of DHA by term infants in the two RCTs remains unclear and two studies were based on infants’ weight. Therefore, it is challenging to definitively conclude whether DHA supplementation has a negligible impact on neurodevelopment. Thirdly, the number of clinical studies examining DHA supplementation in infants alone remains limited, necessitating further research to establish conclusive findings. Lastly, the meta-analysis encompassed numerous parameters for assessing neurological development, making it challenging to synthesize and reconcile their divergent findings.

## Conclusion

The meta-analysis’s supplementation of infants with DHA at certain doses appears beneficial for neurodevelopment, both mental and psychomotor development. However, this is based on a small study and our article has some limitations. Moreover, existing research findings provide some evidence supporting the short-term neurological benefits of DHA supplementation during the peri-pregnancy phase. Conversely, no clear long-term benefits or harms were demonstrated for pregnancies receiving DHA-supplemented formula. The necessity of DHA supplementation in lactating women is uncertain and needs to be explored in more related articles. In the future, it would be beneficial to conduct further investigations to evaluate the effectiveness of DHA supplementation in various populations, especially preterm infants.

## Data availability statement

The original contributions presented in the study are included in the article/Supplementary material, further inquiries can be directed to the corresponding authors.

## Author contributions

RH: Conceptualization, Data curation, Formal analysis, Investigation, Methodology, Software, Validation, Writing – original draft. JX: Data curation, Investigation, Methodology, Software, Writing – original draft. YL: Conceptualization, Funding acquisition, Investigation, Project administration, Resources, Software, Supervision, Validation, Visualization, Writing – original draft, Writing – review & editing. JL: Conceptualization, Funding acquisition, Investigation, Project administration, Software, Supervision, Validation, Visualization, Writing – original draft, Writing – review & editing. YH: Investigation, Project administration, Supervision, Validation, Visualization, writing, Writing – review & editing.
