# Supplementary material for: Could early life DHA supplementation benefit neurodevelopment? A systematic review and meta-analysis
Source: Front Neurol. 2024 Apr 5;15:1295788. doi: 10.3389/fneur.2024.1295788 (PMC11032049; doi:10.3389/fneur.2024.1295788)
Supplement: Supplementary file 1 [file Data_Sheet_1.pdf]

Table 1. Search strategy MEDLINE, EMBASE and Cochrane Library

Search strategy MEDLINE (PubMed)120

((((((((randomized controlled trial[Publication Type]) OR (controlled clinical trial[Publication Type])) OR (randomized[Title/Abstract])) OR (placebo[Title/Abstract])) OR (randomly[Title/Abstract])) OR (trial[Title]))) AND (((((((((((((Nervous System[MeSH Terms]) OR (Nervous Systems[Title/Abstract])) OR (System, Nervous[Title/Abstract])) OR (Systems, Nervous[Title/Abstract])) OR (neural system[Title/Abstract])) OR (neural systems[Title/Abstract])) OR (nerve system[Title/Abstract])) OR (nerve systems[Title/Abstract])) OR (neuronal system[Title/Abstract])) OR (neuronal systems[Title/Abstract])) OR (neurodevelopment[Title/Abstract])) OR (nervous diseases[Title/Abstract])) OR (nervous disease[Title/Abstract]))) AND ((((((((((((((((((((((((Dietary Supplements[MeSH Terms]) OR (Dietary Supplement[Title/Abstract])) OR (Supplements, Dietary[Title/Abstract])) OR (Dietary Supplementations[Title/Abstract])) OR (Supplementations, Dietary[Title/Abstract])) OR (Food Supplementations[Title/Abstract])) OR (Food Supplements[Title/Abstract])) OR (Food Supplement[Title/Abstract])) OR (Supplement, Food[Title/Abstract])) OR (Supplements, Food[Title/Abstract])) OR (Nutraceuticals[Title/Abstract])) OR (Nutraceutical[Title/Abstract])) OR (Nutriceuticals[Title/Abstract])) OR (Nutriceutical[Title/Abstract])) OR (Neutraceuticals[Title/Abstract])) OR (Neutraceutical[Title/Abstract])) OR (Herbal Supplements[Title/Abstract])) OR (Herbal Supplement[Title/Abstract])) OR (Supplement, Herbal[Title/Abstract])) OR (Supplements, Herbal[Title/Abstract])) OR (Supplements[Title/Abstract])) OR (Supplement[Title/Abstract])) OR (Supplementations[Title/Abstract])) OR (Supplementation[Title/Abstract]))) AND ((((((((((((((((((((Docosahexaenoic Acids[MeSH Terms]) OR (Acids, Docosahexaenoic[Title/Abstract])) OR (Docosahexaenoic Acids[Title/Abstract])) OR (Docosahexenoic Acids[Title/Abstract])) OR (Acids, Docosahexenoic[Title/Abstract])) OR (Docosahexaenoic Acid[Title/Abstract])) OR (Acid, Docosahexaenoic[Title/Abstract])) OR (Docosahexaenoic Acid (All-Z Isomer[Title/Abstract]))) OR (Docosahexaenoic Acid Dimer (All-Z Isomer[Title/Abstract]))) OR (Docosahexaenoic Acid, 3,6,9,12,15,18-Isomer[Title/Abstract])) OR (Docosahexaenoic Acid, 4,7,10,13,16,19-(All-Z-Isomer[Title/Abstract]))) OR (Docosahexaenoic Acid, Sodium Salt[Title/Abstract])) OR (Docosahexaenoic Acid, 4,7,10,13,16,19-(All-Z-Isomer), Cesium Salt[Title/Abstract])) OR (Docosahexaenoic Acid, 4,7,10,13,16,19-(All-Z-Isomer), Potassium Salt[Title/Abstract])) OR (Docosahexaenoic Acid, 4,7,10,13,16,19-(Z,Z,Z,Z,Z,E-Isomer[Title/Abstract]))) OR (Docosahexaenoic Acid, 4,7,10,13,16,19-Isomer[Title/Abstract])) OR (Docosahexaenoic Acid, 4,7,10,13,16,19-Isomer, Sodium Salt[Title/Abstract])) OR (Docosahexaenoate[Title/Abstract])) OR (Docosahexaenoic Acid, 4,7,10,13,16,19-(All-Z-Isomer), Cerium Salt[Title/Abstract])) OR (DHA[Title/Abstract])) AND ((humans[Filter]) AND (english[Filter]))

Search strategy EMBASE (Ovid)135

Embase <1974 to 2024 February 07>

1 controlled clinical trial/ or randomized controlled trial/ or controlled study/ or RCT.mp.

2 docosahexaenoic acid.mp. or docosahexaenoic acid/

3 docosahexaenoic acid/ or DHA.mp.

4 nervous system*.mp.

5 neurodevelopment.mp.
6 infants.mp. or infant/
7 2 or 3
8 4 or 5
9 1 and 6 and 7 and 8

Search strategy Cochrane Library47

1 MeSH descriptor: [Docosahexaenoic Acids] explode all trees
2 MeSH descriptor: [Nervous System] explode all trees
3 MeSH descriptor: [Dietary Supplements] explode all trees
4 1 and 2 and 3
