# Supplementary figures and images for: Could early life DHA supplementation benefit neurodevelopment? A systematic review and meta-analysis
Source: Front Neurol. 2024 Apr 5;15:1295788. doi: 10.3389/fneur.2024.1295788 (PMC11032049; doi:10.3389/fneur.2024.1295788)

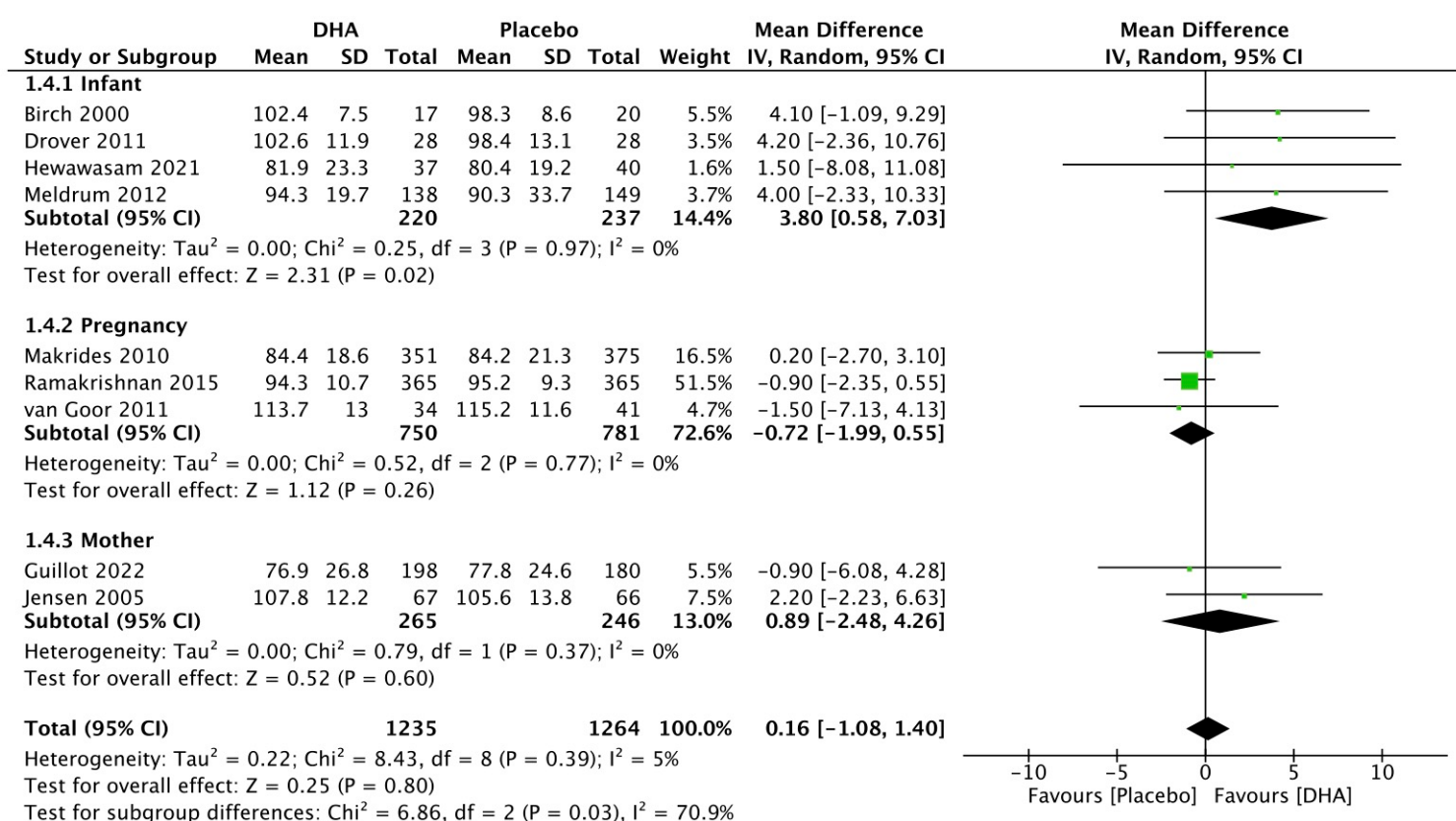

Supplement: Supplementary file 2 [file Data_Sheet_2.pdf]
