# Supplementary material for: Could early life DHA supplementation benefit neurodevelopment? A systematic review and meta-analysis
Source: Front Neurol. 2024 Apr 5;15:1295788. doi: 10.3389/fneur.2024.1295788 (PMC11032049; doi:10.3389/fneur.2024.1295788)
Supplement: Supplementary file 3 [file Data_Sheet_3.pdf]

Study or Subgroup	DHA Mean	DHA SD	DHA Total	Placebo Mean	Placebo SD	Placebo Total	Weight	Mean Difference IV, Random, 95% CI
**3.2.1 Term infant**								
Birch 2000	102.4	7.5	17	98.3	8.6	20	18.1%	4.10 [−1.09, 9.29]
Drover 2011	102.6	11.9	28	98.4	13.1	28	11.3%	4.20 [−2.36, 10.76]
Meldrum 2012	104.7	13.7	138	104	11.1	149	58.0%	0.70 [−2.20, 3.60]
**Subtotal (95% CI)**			**183**			**197**	**87.5%**	**1.86 [−0.50, 4.22]**
Heterogeneity: $Tau^2 = 0.00; Chi^2 = 1.82, df = 2 (P = 0.40); I^2 = 0\%$								
Test for overall effect: Z = 1.54 (P = 0.12)								
**3.2.2 Preterm infant**								
Hewawasam 2021	96.6	13	37	93.2	14.9	40	12.5%	3.40 [−2.83, 9.63]
**Subtotal (95% CI)**			**37**			**40**	**12.5%**	**3.40 [−2.83, 9.63]**
Heterogeneity: Not applicable								
Test for overall effect: Z = 1.07 (P = 0.29)								
**Total (95% CI)**			**220**			**237**	**100.0%**	**2.05 [−0.16, 4.26]**
Heterogeneity: $Tau^2 = 0.00; Chi^2 = 2.03, df = 3 (P = 0.57); I^2 = 0\%$								
Test for overall effect: Z = 1.82 (P = 0.07)								
Test for subgroup differences: $Chi^2 = 0.21, df = 1 (P = 0.65), I^2 = 0\%$								

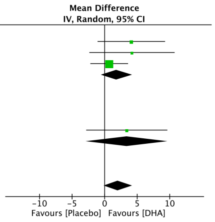

Study or Subgroup	DHA Mean	DHA SD	DHA Total	Placebo Mean	Placebo SD	Placebo Total	Weight	Mean Difference IV, Random, 95% CI
**3.1.1 Term infant**								
Birch 2000	102.4	7.5	17	98.3	8.6	20	38.6%	4.10 [−1.09, 9.29]
Drover 2011	102.6	11.9	28	98.4	13.1	28	24.2%	4.20 [−2.36, 10.76]
Meldrum 2012	94.3	19.7	138	90.3	33.7	149	25.9%	4.00 [−2.33, 10.33]
**Subtotal (95% CI)**			**183**			**197**	**88.7%**	**4.10 [0.68, 7.52]**
Heterogeneity: $Tau^2 = 0.00; Chi^2 = 0.00, df = 2 (P = 1.00); I^2 = 0\%$								
Test for overall effect: Z = 2.35 (P = 0.02)								
**3.1.2 Preterm infant**								
Hewawasam 2021	81.9	23.3	37	80.4	19.2	40	11.3%	1.50 [−8.08, 11.08]
**Subtotal (95% CI)**			**37**			**40**	**11.3%**	**1.50 [−8.08, 11.08]**
Heterogeneity: Not applicable								
Test for overall effect: Z = 0.31 (P = 0.76)								
**Total (95% CI)**			**220**			**237**	**100.0%**	**3.80 [0.58, 7.03]**
Heterogeneity: $Tau^2 = 0.00; Chi^2 = 0.25, df = 3 (P = 0.97); I^2 = 0\%$								
Test for overall effect: Z = 2.31 (P = 0.02)								
Test for subgroup differences: $Chi^2 = 0.25, df = 1 (P = 0.62), I^2 = 0\%$								

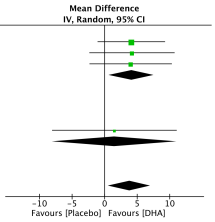

Study or Subgroup	DHA Mean	DHA SD	DHA Total	Placebo Mean	Placebo SD	Placebo Total	Weight	Mean Difference IV, Random, 95% CI
**3.3.1 Term infant**								
Birch 2000	99	4	17	98.3	8.6	20	16.4%	0.70 [−3.52, 4.92]
Drover 2011	104.1	11.3	28	102	6.3	28	12.8%	2.10 [−2.69, 6.89]
Meldrum 2012	105.4	8.8	138	103.4	11.1	149	54.9%	2.00 [−0.31, 4.31]
**Subtotal (95% CI)**			**183**			**197**	**84.1%**	**1.76 [−0.10, 3.63]**
Heterogeneity: $Tau^2 = 0.00; Chi^2 = 0.30, df = 2 (P = 0.86); I^2 = 0\%$								
Test for overall effect: Z = 1.85 (P = 0.06)								
**3.3.2 Preterm infant**								
Hewawasam 2021	98.8	8.9	37	95.9	10.3	40	15.9%	2.90 [−1.39, 7.19]
**Subtotal (95% CI)**			**37**			**40**	**15.9%**	**2.90 [−1.39, 7.19]**
Heterogeneity: Not applicable								
Test for overall effect: Z = 1.32 (P = 0.19)								
**Total (95% CI)**			**220**			**237**	**100.0%**	**1.94 [0.23, 3.65]**
Heterogeneity: $Tau^2 = 0.00; Chi^2 = 0.53, df = 3 (P = 0.91); I^2 = 0\%$								
Test for overall effect: Z = 2.22 (P = 0.03)								
Test for subgroup differences: $Chi^2 = 0.23, df = 1 (P = 0.63), I^2 = 0\%$								

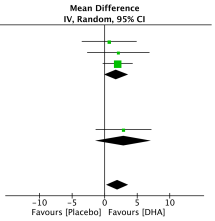

Outcome indicators of infant from top to bottom of the forest plots are MDI (language scores conversion), MDI (cognitive scores conversion) and PDI.

Study or Subgroup	DHA Mean	DHA SD	DHA Total	Placebo Mean	Placebo SD	Placebo Total	Weight	Mean Difference IV, Random, 95% CI
Makrides 2010	96.5	13.6	351	97.9	15.3	375	31.0%	−1.40 [−3.50, 0.70]
Ramakrishnan 2015	94.3	10.7	365	95.2	9.3	365	64.7%	−0.90 [−2.35, 0.55]
van Goor 2011	113.7	13	34	115.2	11.6	41	4.3%	−1.50 [−7.13, 4.13]
**Total (95% CI)**			**750**			**781**	**100.0%**	**−1.08 [−2.25, 0.09]**
Heterogeneity: $Tau^2 = 0.00; Chi^2 = 0.17, df = 2 (P = 0.92); I^2 = 0\%$								
Test for overall effect: Z = 1.81 (P = 0.07)								

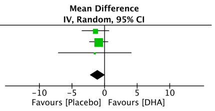

Study or Subgroup	DHA Mean	DHA SD	DHA Total	Placebo Mean	Placebo SD	Placebo Total	Weight	Mean Difference IV, Random, 95% CI
Makrides 2010	84.4	18.6	351	84.2	21.3	375	19.0%	0.20 [−2.70, 3.10]
Ramakrishnan 2015	94.3	10.7	365	95.2	9.3	365	75.9%	−0.90 [−2.35, 0.55]
van Goor 2011	113.7	13	34	115.2	11.6	41	5.1%	−1.50 [−7.13, 4.13]
**Total (95% CI)**			**750**			**781**	**100.0%**	**−0.72 [−1.99, 0.55]**
Heterogeneity: $Tau^2 = 0.00; Chi^2 = 0.52, df = 2 (P = 0.77); I^2 = 0\%$								
Test for overall effect: Z = 1.12 (P = 0.26)								

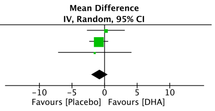

Study or Subgroup	DHA Mean	DHA SD	DHA Total	Placebo Mean	Placebo SD	Placebo Total	Weight	Mean Difference IV, Random, 95% CI
Makrides 2010	102.6	10.2	351	101.8	12.6	375	41.2%	0.80 [−0.86, 2.46]
Ramakrishnan 2015	93	8.9	365	93.3	9.8	365	48.4%	−0.30 [−1.66, 1.06]
van Goor 2011	95.8	11.4	34	91.7	8.3	41	10.4%	4.10 [−0.50, 8.70]
**Total (95% CI)**			**750**			**781**	**100.0%**	**0.61 [−0.99, 2.21]**
Heterogeneity: $Tau^2 = 0.89; Chi^2 = 3.72, df = 2 (P = 0.16); I^2 = 46\%$								
Test for overall effect: Z = 0.75 (P = 0.45)								

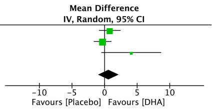

Outcome indicators of pregnancy from top to bottom of the forest plots are MDI (language scores conversion), MDI (cognitive scores conversion) and PDI.

Study or Subgroup	DHA Mean	DHA SD	DHA Total	Placebo Mean	Placebo SD	Placebo Total	Weight	Mean Difference IV, Random, 95% CI
Guillot 2022	89.5	16.1	190	87.4	16.7	176	63.4%	2.10 [−1.27, 5.47]
Jensen 2005	107.8	12.2	67	105.6	13.8	66	36.6%	2.20 [−2.23, 6.63]
**Total (95% CI)**			**257**			**242**	**100.0%**	**2.14 [−0.54, 4.82]**
Heterogeneity: $Tau^2 = 0.00; Chi^2 = 0.00, df = 1 (P = 0.97); I^2 = 0\%$								
Test for overall effect: Z = 1.56 (P = 0.12)								

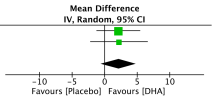

Study or Subgroup	DHA Mean	DHA SD	DHA Total	Placebo Mean	Placebo SD	Placebo Total	Weight	Mean Difference IV, Random, 95% CI
Guillot 2022	76.9	26.8	198	77.8	24.6	180	42.2%	−0.90 [−6.08, 4.28]
Jensen 2005	107.8	12.2	67	105.6	13.8	66	57.8%	2.20 [−2.23, 6.63]
**Total (95% CI)**			**265**			**246**	**100.0%**	**0.89 [−2.48, 4.26]**
Heterogeneity: $Tau^2 = 0.00; Chi^2 = 0.79, df = 1 (P = 0.37); I^2 = 0\%$								
Test for overall effect: Z = 0.52 (P = 0.60)								

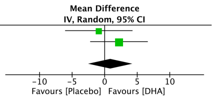

Study or Subgroup	DHA Mean	DHA SD	DHA Total	Placebo Mean	Placebo SD	Placebo Total	Weight	Mean Difference IV, Random, 95% CI
Guillot 2022	92.7	14.5	189	91.5	13.8	167	56.4%	1.20 [−1.74, 4.14]
Jensen 2005	116.8	15.2	68	108.4	18.9	65	43.6%	8.40 [2.56, 14.24]
**Total (95% CI)**			**257**			**232**	**100.0%**	**4.34 [−2.66, 11.34]**
Heterogeneity: $Tau^2 = 20.35; Chi^2 = 4.65, df = 1 (P = 0.03); I^2 = 78\%$								
Test for overall effect: Z = 1.22 (P = 0.22)								

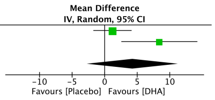

Outcome indicators of mother from top to bottom of the forest plots are MDI (language scores conversion), MDI (cognitive scores conversion) and PDI.
